# Supplementary material for: Mapping the effective coverage of modern contraceptive services in Ethiopia
Source: PLoS One. 2025 Jul 7;20(7):e0327581. doi: 10.1371/journal.pone.0327581 (PMC12233249; doi:10.1371/journal.pone.0327581)
Supplement: S1 Table — Quality indicator variables for modern contraceptive services (SPA 2021–2022). (DOCX) [file pone.0327581.s001.docx]

S1 Table: Quality indicator variables for modern contraceptive services (SPA 2021-2022)

|  | Structural | Facility recode |
| --- | --- | --- |
| 1 | Received any training on FP | sf1322a |
| 2 | Received any training on FP - other providers | sf1322b |
| 3 | Guidelines: national family planning guidelines | v327a |
| 4 | Guidelines: other family planning guidelines | v327x |
| 5 | Family planning exam: guidelines for standard precautions | v331u |
| 6 | Avail/valid: (3-monthly) injectable progestin only (depo provera) | lv303e |
| 7 | Avail/valid: iud | lv303k |
| 8 | Avail/valid: implant | lv303g |
| 9 | Avail/valid: emergency contraceptive pill | lv303l |
| 10 | Avail/valid: progesterone only pill | lv303b |
| 11 | Avail/valid: combined oral pill | lv303a |
| 12 | Avail/valid: male condom | lv303i |
| 13 | Avail/valid: emergency contraceptive pill | lv303l |
| 14 | HIV RTD kit available in fp service area | v344 |
| 15 | Family planning exam: any running water | v331a1 |
| 16 | Family planning exam: soap | v331c |
| 17 | Family planning exam: sharps container | v331f |
| 18 | Family planning exam: clean/sterile latex gloves | v331g |
| 19 | Family planning exam: disinfectant | v331j |
| 20 | Family planning exam: auto-disable syringes with needles/single-use standard dis | v331l |
| 21 | Privacy for family planning exam | v330 |
| 22 | Supplies: samples of fp methods | v325a |
| 23 | Contraceptives stored in area protected from sunlight | lv320a |
| 24 | Contraceptives stored in area protected from water | lv320e |
| 25 | Contraceptives stored in area well ventilated | lv320f |
|  | **Process** | **Facility recode** |
| 1 | Offer: combined oral pill | v303a |
| 2 | Offer: progestin-only pill | v303b |
| 3 | Offer: injectable progestin-only (depo Provera 3-monthly) | v303e |
| 4 | Offer: male | v303i |
| 5 | Offer: female condom | v303j |
| 6 | Offer: iucd | v303k |
| 7 | Offer: emergency contraceptive pill | v303l |
| 8 | Offer: male sterilization (vasectomy) | v303q |
| 9 | Offer: female sterilization (tubal ligation) | v303r |
| 10 | Offer: implant (jadelle, implanon or sino-implant) | v303g |
| 11 | Offer: counseling on natural methods (periodic abstinence) | v303o |
| 12 | Provider routinely treats stis | v308 |
| 13 | Routine tx: counseling about family planning | v409b |
| 14 | Routine: weigh client | v307a |
| 15 | Routine: take blood pressure | v307b |
| 16 | Routine: group health sessions | v307c |
|  |  | **FP recode** |
| 17 | Client privacy: ensure visual privacy | c304a |
| 18 | Client privacy: ensure auditory privacy | c304b |
| 19 | Client privacy: assure client orally of confidentiality | c304c |
| 20 | Issues discussed: risk of stis | c305c |
| 21 | Issues discussed: use condoms with other method to avoid pregnancy and stis/hiv | c305e |
| 22 | Issues discussed: partner's attitude toward fp | c305a |
| 23 | Issues discussed: use of condoms to prevent STIs/HIV | c305d |
| 24 | Issues discussed: ask client about questions/concerns regarding current method | c305f |
| 25 | Issues discussed: client expressed concerns or asked questions | c305g |
| 26 | during consult, provider: explained how to use method | c362a |
| 27 | during consult, provider: talked about possible side effects | c362b |
| 28 | during consult, provider: advised what to do if problems occurred | c362c |
| 29 | during consult, provider: told when to return for follow-up | c362d |
| 30 | Provider talked about any considered methods | c358 |
|  | **Outcome** | **FP recode** |
| 1 | Friendliness of the provider | sx401 |
| 2 | Amount of time provider spent with client | sx402 |
| 3 | How clearly provider communicated information to client | sx403 |
| 4 | Wait time before client saw a provider | sx404 |
| 5 | Likelihood of recommending family member/ friend getting services | sx406 |
| 6 | The provider was friendly and warm towards me | sx407 |
| 7 | The care provider was sympathetic and concerned about me | sx408 |
| 8 | The provider did not always understand the way i felt inside | sx409 |
| 9 | Confidence client will receive effective treatment from health system | sx411 |
| 10 | Client felt could trust care provider during today’s consultation | sx412 |
| 11 | Client rating for knowledge & competence of providers at this facility during | sx414 |
| 12 | Client problem during visit: visual privacy | c502e |
| 13 | Client problem during visit: auditory privacy | c502f |
| 14 | Client problem during visit: availability of medicines at facility | c502g |
| 15 | Client problem during visit: hours of service | c502h |
| 16 | Client problem during visit: days of service | c502i |
| 17 | Client problem during visit: cleanliness of facility | c502j |
