# Supplementary material for: Mapping the effective coverage of modern contraceptive services in Ethiopia
Source: PLoS One. 2025 Jul 7;20(7):e0327581. doi: 10.1371/journal.pone.0327581 (PMC12233249; doi:10.1371/journal.pone.0327581)
Supplement: S2 Table — Effective coverage of modern contraceptive services in Ethiopia 2019 EMDHS, with administrative data linkage. (DOCX) [file pone.0327581.s002.docx]

S2 Table: Effective coverage of modern contraceptive services in Ethiopia 2019 EMDHS, with administrative data linkage.

| Region | Crude coverage (95% CI) | EC % (95% CI) | CC-EC (% point) |
| --- | --- | --- | --- |
| Addis Ababa | 26.52 (23.33, 29.71) | 19.55 (18.83, 20.26) | 6.97 |
| Afar | 12.54 (9.61, 15.47) | 7.69 (7.21, 8.17) | 4.85 |
| Amhara | 36.41 (32.96, 39.86) | 25.09 (24.44, 25.75) | 11.32 |
| Benishangul-Gumuz | 28.20 (24.39, 32.02) | 18.64 (17.46, 19.81) | 9.56 |
| Dire Dawa | 19.99 (16.94, 23.03) | 13.97 (13.24, 14.70) | 6.02 |
| Gambela | 25.14 (20.84, 29.44) | 14.79 (14.14, 15.43) | 10.35 |
| Harari | 21.71 (18.56, 24.86) | 15.23 (13.41, 17.05) | 6.48 |
| Oromia | 29.90 (26.90, 33.00) | 20.00 (19.52, 20.44) | 9.9 |
| SNNP | 33.73 (30.12, 37.39) | 22.91 (22.44, 23.37) | 10.82 |
| Somali | 3.25 (1.54, 4.97) | 1.96 (1.79, 2.13) | 1.29 |
| National level | 28.96 (28.36, 29.56) | 19.5 (19.03,19.99) | 9.45 |
| SNNPE: Southern Nations, Nationalities and Peoples; EC: Effective coverage; CC: Crude coverage | | | |
