# Supplementary material for: Mapping the effective coverage of modern contraceptive services in Ethiopia
Source: PLoS One. 2025 Jul 7;20(7):e0327581. doi: 10.1371/journal.pone.0327581 (PMC12233249; doi:10.1371/journal.pone.0327581)
Supplement: Attachment — Submitted filename: PONE-D-24-60655 ME 20250312.pdf [file pone.0327581.s005.pdf]

PLOS ONE

Spatial distribution of effective coverage of modern contraceptive service in Ethiopia

--Manuscript Draft--

Manuscript Number:	PONE-D-24-60655
Article Type:	Research Article
Full Title:	Spatial distribution of effective coverage of modern contraceptive service in Ethiopia
Short Title:	Spatial distribution of effective coverage of modern contraceptive service
Corresponding Author:	Samrawit Alemu, MSc<br>Debre Markos University<br>Debre Markos, ETHIOPIA
Keywords:	
Abstract:	Introduction<br><br>Given its significant contribution to the reduction of maternal and infant mortality and morbidity, family planning is commonly regarded as a major intervention towards the achievement of national and international goals. Low service quality results in poor effective coverage and reduces the potential health benefit from using these services. However, the current effective coverage of modern contraceptive in Ethiopia is not known.<br><br>Methods<br><br>We used the ESPA's family planning recode, the health facility recode, and the IR file (individual record) from the EDHS. Next, region was used to link the ESPA and EDHS 2019 data. Stata 17 were used for analysis. The score of each component was calculated as proportion of recommended clinical actions done in family planning to the total number of indices in each component. The effective coverage of modern contraceptive use was calculated by multiplying the quality of the health facility with the modern contraceptive use. We use ArcGIS 10.7 software to analyze the spatial distribution after determining the effective coverage of modern contraceptives. A measure of spatial autocorrelation called the Global Moran's I statistic was used to assess the spatial distribution of modern contraceptives. A hot spot analysis was performed using the Getis-Ord Gi* statistic, which successfully identified noteworthy clusters of modern contraception that were high (hot places) and low (cold spots). The kriging spatial interpolation approach was used to forecast the effective coverage of modern contraceptives in unsampled regions of Ethiopia based on sampled clusters.<br><br>Result<br><br>The national effective coverage of modern contraceptive service is 19.51% (95% CI: 19.03, 19.99) with a large variation in different regions, ranging from 7.69% in Afar to 25.09% in Amhara. The spatial distribution analysis showed that facilities found in Amhara, Addis Ababa and the Northern part of SNNPE had higher effective coverage. There were hot and cold spots areas for effective coverage of modern contraceptive in Ethiopia. Addis Ababa, Amhara, North SNNP, and Northern Oromia had hotspots for effective coverage of modern contraceptive. On the other hand, Afar, Gambela, Dire Dawa, Harrarie and Somali had cold spots for Effective coverage of modern contraceptive.<br><br>Conclusion<br><br>The study found that, Ethiopia had a poor effective coverage of modern contraceptives services. Somalia and Afar regions had less effective coverage of modern contraceptive service. The modern contraceptive service quality were very low and mainly process component were unsatisfactory. Private facilities and facilities in rural areas had lower-quality modern contraceptive services. Policymakers and responsible bodies should focus on improving the quality of the facilities in rural areas and the facilities in Gambela, Somali and Afar.

Powered by Editorial Manager® and ProduXion Manager® from Aries Systems Corporation

Order of Authors:	Samrawit Alemu, MSc
	Molla Yigzaw Birhanu
	Mekdes Tamiru Yizengaw
	Melaku Birhanu Alemu
**Additional Information:**	
**Question**	**Response**
**Financial Disclosure**<br><br>Enter a financial disclosure statement that describes the sources of funding for the work included in this submission. Review the submission guidelines for detailed requirements. View published research articles from *PLOS ONE* for specific examples.<br><br>This statement is required for submission and **will appear in the published article** if the submission is accepted. Please make sure it is accurate.<br><br>**Funded studies**<br>Enter a statement with the following details:<br>• Initials of the authors who received each award<br>• Grant numbers awarded to each author<br>• The full name of each funder<br>• URL of each funder website<br>• Did the sponsors or funders play any role in the study design, data collection and analysis, decision to publish, or preparation of the manuscript?<br><br>Did you receive funding for this work?	The author(s) received no specific funding for this work.
**Competing Interests**<br><br>Use the instructions below to enter a competing interest statement for this submission. On behalf of all authors, disclose any competing interests that could be perceived to bias this work—acknowledging all financial support and any other relevant financial or non-financial competing interests.	The authors declare that they have no competing interests.

Powered by Editorial Manager® and ProduXion Manager® from Aries Systems Corporation

This statement is required for submission and **will appear in the published article** if the submission is accepted. Please make sure it is accurate and that any funding sources listed in your Funding Information later in the submission form are also declared in your Financial Disclosure statement.<br><br>View published research articles from *PLOS ONE* for specific examples.<br><br>**NO authors have competing interests**<br><br>Enter: *The authors have declared that no competing interests exist.*<br><br>**Authors with competing interests**<br><br>Enter competing interest details beginning with this statement:<br><br>*I have read the journal's policy and the authors of this manuscript have the following competing interests: [insert competing interests here]*<br><br>* typeset	
**Ethics Statement**<br><br>Enter an ethics statement for this submission. This statement is required if the study involved:<br><br>• Human participants<br>• Human specimens or tissue<br>• Vertebrate animals or cephalopods<br>• Vertebrate embryos or tissues<br>• Field research<br><br>Write "N/A" if the submission does not require an ethics statement.<br><br>General guidance is provided below. Consult the submission guidelines for detailed instructions. **Make sure that all information entered here is included in the Methods section of the manuscript.**	Patient and public involvement: Patients and/or the public were not involved in the design, or conduct, or reporting, or dissemination plans of this research.

Powered by Editorial Manager® and ProduXion Manager® from Aries Systems Corporation

**Format for specific study types**<br><br>**Human Subject Research (involving human participants and/or tissue)**<br>• Give the name of the institutional review board or ethics committee that approved the study<br>• Include the approval number and/or a statement indicating approval of this research<br>• Indicate the form of consent obtained (written/oral) or the reason that consent was not obtained (e.g. the data were analyzed anonymously)<br><br>**Animal Research (involving vertebrate animals, embryos or tissues)**<br>• Provide the name of the Institutional Animal Care and Use Committee (IACUC) or other relevant ethics board that reviewed the study protocol, and indicate whether they approved this research or granted a formal waiver of ethical approval<br>• Include an approval number if one was obtained<br>• If the study involved *non-human primates*, add *additional details* about animal welfare and steps taken to ameliorate suffering<br>• If anesthesia, euthanasia, or any kind of animal sacrifice is part of the study, include briefly which substances and/or methods were applied<br><br>**Field Research**<br><br>Include the following details if this study involves the collection of plant, animal, or other materials from a natural setting:<br>• Field permit number<br>• Name of the institution or relevant body that granted permission	
**Data Availability**<br><br>Authors are required to make all data underlying the findings described fully available, without restriction, and from the time of publication. PLOS allows rare exceptions to address legal and ethical concerns. See the PLOS Data Policy and FAQ for detailed information.	Yes - all data are fully available without restriction

A Data Availability Statement describing where the data can be found is required at submission. Your answers to this question constitute the Data Availability Statement and **will be published in the article**, if accepted.<br><br>**Important:** Stating 'data available on request from the author' is not sufficient. If your data are only available upon request, select 'No' for the first question and explain your exceptional situation in the text box.<br><br>Do the authors confirm that all data underlying the findings described in their manuscript are fully available without restriction?	
**Describe where the data may be found in full sentences. If you are copying our sample text, replace any instances of XXX with the appropriate details.**<br><br>• If the data are **held or will be held in a public repository**, include URLs, accession numbers or DOIs. If this information will only be available after acceptance, indicate this by ticking the box below. For example: *All XXX files are available from the XXX database (accession number(s) XXX, XXX.*).<br>• If the data are all contained **within the manuscript and/or Supporting Information files**, enter the following: *All relevant data are within the manuscript and its Supporting Information files.*<br>• If neither of these applies but you are able to provide **details of access elsewhere**, with or without limitations, please do so. For example:<br><br>*Data cannot be shared publicly because of [XXX]. Data are available from the XXX Institutional Data Access / Ethics Committee (contact via XXX) for researchers who meet the criteria for access to confidential data.*<br><br>*The data underlying the results presented in the study are available*	The Ethiopian demographic health survey (DHS) dataset is available to the general public upon request from the Measure DHS website http://www.measuredhs.com.

from (include the name of the third party and contact information or URL).<br>• This text is appropriate if the data are owned by a third party and authors do not have permission to share the data.<br><br>* typeset	
Additional data availability information:	

Powered by Editorial Manager® and ProduXion Manager® from Aries Systems Corporation

Manuscript

Click here to access/download;Manuscript;EC_modern contraceptive (Autosaved).docx

Spatial distribution of effective coverage of modern contraceptive service in Ethiopia

Samrawit Birhanu Alemu[1*], Molla Yigzaw Birhanu[1], Mekdes Tamiru Yizengaw [2], Melaku Birhanu Alemu[4,5]

[1] Department of Public Health, Debre Markos University, Debre Markos, Ethiopia

[2] St. Lideta Health Science College, Addis Ababa, Ethiopia

[3] School of Medicine, College of Medicine and Health Science, University of Gondar, Gondar, Ethiopia

[4] Department of Health Systems and Policy, Institute of Public Health, University of Gondar, Gondar, Ethiopia

[5] Curtin School of Population Health, Curtin University, Perth, Western Australia, Australia

Email addresses:

SBA: samribtm@gmail.com

MYB: mollayigzaw33@gmail.com

MTY: mekdesmela@gmail.com

YG: yuanyuan.gu@mq.edu.au

MBA: melakubt@gmail.com

***Corresponding Author:**

Samrawit Birhanu Alemu

samribtm@gmail.com

ORCID: https://orcid.org/0009-0001-3905-8889

Abstract

Introduction: Given its significant contribution to the reduction of maternal and infant mortality and morbidity, family planning is commonly regarded as a major intervention towards the achievement of national and international goals. Low service quality results in poor effective coverage and reduces the potential health benefit from using these services. However, the current effective coverage of modern contraceptive in Ethiopia is not known.

Methods: We used the ESPA's family planning recode, the health facility recode, and the IR file (individual record) from the EDHS. Next, GPS was used to link the ESPA and EDHS 2019 data. Stata 17 were used for analysis. The score of each component was calculated as proportion of recommended clinical actions done in family planning to the total number of indices in each component. The effective coverage of modern contraceptive use was calculated by multiplying the quality of the health facility with the modern contraceptive use. We use ArcGIS 10.7 software to analyze the spatial distribution after determining the effective coverage of modern contraceptives. A measure of spatial autocorrelation called the Global Moran's I statistic was used to assess the spatial distribution of modern contraceptives. A hot spot analysis was performed using the Getis-Ord Gi* statistic, which successfully identified noteworthy clusters of modern contraception that were high (hot places) and low (cold spots). The kriging spatial interpolation approach was used to forecast the effective coverage of modern contraceptives in unsampled regions of Ethiopia based on sampled clusters.

Result: The national effective coverage of modern contraceptive service is 19.51% (95% CI: 19.03, 19.99) with a large variation in different regions, ranging from 7.69% in Afar to 25.09% in Amhara. The spatial distribution analysis showed that facilities found in Amhara, Addis Ababa and the Northern part of SNNPE had higher effective coverage. There were hot and cold spots areas for effective coverage of modern contraceptive in Ethiopia. Addis Ababa, Amhara, North SNNP, and Northern Oromia had hotspots for effective coverage of modern contraceptive. On the other hand, Afar, Gambela, Dire Dawa, Harrarie and Somali had cold spots for Effective coverage of modern contraceptive.

Conclusion: The study found that, Ethiopia had a poor effective coverage of modern contraceptives services. Somalia and Afar regions had less effective coverage of modern contraceptive service. Addis Ababa, Amhara, North SNNP, and Northern Oromia had hotspots for effective coverage of modern contraceptive. On the other hand, Afar, Gambela, Dire Dawa, Harrarie and Somali had cold spots for Effective coverage of modern contraceptive.Policymakers and responsible bodies should focus on improving the quality of the facilities in rural areas and the facilities in Gambela, Somali and Afar.

Introduction

Given its significant contribution to the reduction of maternal and infant mortality and morbidity, family planning is commonly regarded as a major intervention towards the achievement of national and international goals [1]. Family planning techniques fall into two categories: traditional techniques, such as rhyme and withdrawal techniques, and modern techniques, which include condoms, sterilization, tablets, injectable, and intrauterine devices (IUDs). Particularly in low- and middle-income nations, modern contraceptive methods have proven effective in reducing fertility and moderating population increase. [2-5].

Globally, it is estimated that 76% of 1.1 billion women who need family planning were using modern contraceptives [6, 7]. An estimated 190 million reproductive age group women who want to prevent pregnancy do not use any contraceptive method. Therefore, this indicates the prevalence of unmet need for modern contraceptives among married or in-union women is around 10% [7].

Every year, more than 2 million newborns and children's lives have been found to be saved through birth spacing by increased use of modern contraceptive methods [8]. Therefore, improved access to family planning improves maternal health and benefits child survival [9]. Furthermore, enhancing family planning has the potential to avert 32% of maternal mortality and around 10% of childhood deaths and reduce poverty and hunger in countries with high birth rates [10].

In both developed and developing nations, quality is the primary factor and the primary predictor of achieving the sustainable development goals [11]. Quality of care is a complex concept that requires a wide range of information to be measured, including facility readiness, processes of care and users' experiences, and effects of care, to conceptualize quality from inputs, processes, and outcome perspectives, the Donabedian model of quality health care is widely used [12].

Maternal mortality rates that continue to remain high may be caused by inadequate care, which was not reflected in the basic coverage measures. [13]. Low service quality results in poor effective coverage and reduces the potential health benefit from using these services, counteracting the relatively high crude coverage rates for modern contraceptive [14]. Because crude coverage cannot assess the degree of adherence to care standards or the manner in which services were delivered, it only evaluates access to services and does not take quality of care into consideration. [15, 16].

Despite significant growth in the number of health facilities delivering family planning, the quality of these services remains persistently inadequate in Ethiopia [14]. There were various studies conducted on the coverage of modern contraceptives [17-19]. However the quality adjusted coverage of modern contraceptive service has not been studied. So, this study will assess the effective coverage of modern contraceptive in Ethiopia. Policy makers will use the findings to improve the quality of service and quality adjusted service coverage.

Methods

Study area and data source

Ethiopia is a federal state with two city administrations and nine sub-regions that is situated on the Horn of Africa. The 2019 mini EDHS data, which was intended to offer estimates of important indicators for the nation as a whole, for urban and rural areas separately, for each of the nine regions, and for the two administrative cities, and SPA 2020–2021, which is the second facility assessment that gathers information on service availability and quality of care, was used in this study. The EDHS holds data that is publicly available via Measure DHS at https://dhsprogram.com/data/ available-datasets.cfm.

Measure of variable

Coverage variable

The coverage measures were taken from the mini EDHS 2019. Modern contraceptive was coded as "yes" for those who use modern contraceptives and "no" for those who did not use any contraceptive and use traditional methods [20].

Quality of care variables

There are three dimensions used to measure the quality of service, these are structure, process, and outcome [12]. The quality measures were taken from the facility recode and family planning recode, ESPA 2020-2021. We use modern contraceptive recommended services which were used for quality measure from the national guideline for family planning in Ethiopia [21]. Each indicators were recoded as “yes” and “no” [22]. A total of 72 indicators were used, where 25 structural, 30 process, 17 were outcome indicators (See supplementary file).

Data analysis

Stata 17 were used for analysis. We used the ESPA's family planning recode, the health facility recode, and the IR file (individual record) from the EDHS with 7618 total sample size of reproductive age group women. While the ESPA's GPS data was linked to the facility and family planning records, the DHS survey's GPS data was linked to the individual records. The "geonear" STATA command was used to link the EDHS and ESPA data using GPS coordinates using the nearest adjacent approaches. The Tigray region was not included in the analysis as there were no records for it in the ESPA. Only 648 facilities which had exit interview records for family planning were included in the study. A DHS statistics guide was used to clean up the data and handle any missing numbers. To restore the survey's representativeness, the data were weighted using sample weight.

The quality of modern contraceptive was calculated using additive weighting methods, which is weighting each quality component based on the number of service quality indicators used [23]. Therefore, the structural component constituted 34.7% (25/72*100) of the total, process components had 41.7% (30/72*100), and the outcome component had 23.6% (17/72*100). The score of each component was calculated as proportion of recommended clinical actions done in family planning to the total number of indices in each component.

$$Structural\ component = \frac{Total\ number\ of\ \text{"yes"}\ responses\ in\ the\ componenent}{Total\ number\ of\ parameter\ in\ the\ structure\ component} * Weight$$

$$Process\ component = \frac{Total\ number\ of\ \text{"yes"}\ responses\ in\ the\ component}{Total\ number\ of\ parameter\ in\ the\ process\ component} * Weight$$

$$Outcome\ component = \frac{Total\ number\ of\ \text{"yes"}\ responses\ in\ the\ component}{Total\ number\ of\ parameter\ in\ the\ process\ component} * Weight$$

The effective coverage of modern contraceptive use was calculated by multiplying the quality of the health facility with the modern contraceptive use [16, 24].

Effective coverage = Quality of the health service * crude coverage

The facility's characteristics as well as the respondents' socioeconomic and demographic characteristics were described using descriptive statistics. Lastly, tables and graphs were used to present the results.

We use ArcGIS 10.7 software to analyze the spatial distribution after determining the effective coverage of modern contraceptives. A measure of spatial autocorrelation called the Global Moran's I statistic was used to assess the spatial distribution of modern contraceptives that work in Ethiopia. The distribution of modern contraceptives across regions can be evaluated using this method to determine if it follows a spatial pattern or is random. With a positive Global Moran's I value, the analysis's findings suggested that Ethiopia's coverage of modern contraceptives is not dispersed randomly but rather is concentrated in particular places, with some showing higher or lower coverage than would be predicted by chance [25].

A hot spot analysis was performed using the Getis-Ord Gi* statistic, which successfully identified noteworthy clusters of modern contraception that were high (hot places) and low (cold spots). In order to identify regions where modern contraceptive efforts are particularly successful or where coverage gaps are noticeable, this spatial statistical method is frequently used to find trends in geographic data [26]. The kriging spatial interpolation approach was used to forecast the effective coverage of modern contraceptives in unsampled regions of Ethiopia based on sampled clusters [27]. Kriging is a geostatistical technique that uses spatial autocorrelation and data from neighboring sampled points to estimate values at unmeasured places. In areas where direct data collecting has not been carried out, this method guarantees more precise and trustworthy forecasts of the coverage of modern contraceptives [28].

Ethics approval and consent to participate

The Ethiopian demographic health survey (DHS) dataset is available to the general public upon request from the Measure DHS website http://www.measuredhs.com. We submitted a request to the DHS by briefly stating the objectives of this analysis and thereafter received permission to download the maternal record dataset.

Patient and public involvement: Patients and/or the public were not involved in the design, or conduct, or reporting, or dissemination plans of this research.

Results

A total of weighted 7618 reproductive age women were included. Of these, 42.25% were below 24 year, 36.01% of women were between 25-35 years and 21.74% were above age 36. Furthermore, the vast majority of participants (67.88%) were from rural areas (Table 1).

Table 1: Socioeconomic and demographic characteristics of participants in 2019 (n=7618)

**Variables**		**Number**	**Frequency%**
Age	15-24	3,219	42.25
	25-35	2,743	36.01
	36-49	1,656	21.74
Marital status	Unmarried	2,744	36.01
	Married	4,875	63.99
Residence	Rural	5,171	67.88
	Urban	2,447	32.12
Religion	Orthodox	2,915	38.27
	Muslim	2,342	30.75
	Protestant	2,238	29.37
	Others	123	1.61
Region	Afar	76	0.99
	Amhara	1915	25.14
	Oromia	3070	40.3
	Somali	362	4.75
	Benshangule	90	1.19
	SNNPR	1574	20.66
	Gambela	37	0.49
	Harari	24	0.31
	Addis Abeba	411	5.4
	Dire Dawa	59	0.77
Education	No education	3,077	40.39
	Primary education	3,255	42.73

	Secondary education	905	11.89
	Higher	381	5
Wealth	Poorest	1,182	15.51
	Poorer	1,416	18.59
	Middle	1,468	19.27
	Richer	1,686	22.13
	Richest	1,866	24.5

A total of 648 health facilities were included. The majority of the facilities were hospitals (43.98%). Most of the facilities were governmental facilities (82.41%) (Table 2).

Table 2: Health facilities characteristics Ethiopian SPA 2020-2021(n=648)

**Variable**		**Frequency**	**Percentage**
**Facility type**	Hospital	285	43.98
	Health centre	209	32.25
	Health post	64	9.88
	Clinic	90	13.89
**Area**	Urban	374	57.72
	Rural	274	42.28
**Managing authority**	Government	534	82.41
	Private	100	15.43
	NGO	14	2.16

Crude coverage of modern contraceptive

The crude coverage of modern contraceptive services was 28.96%. And it ranges from 3.25% in Somali to 36.41% in Amhara (Figure 1).

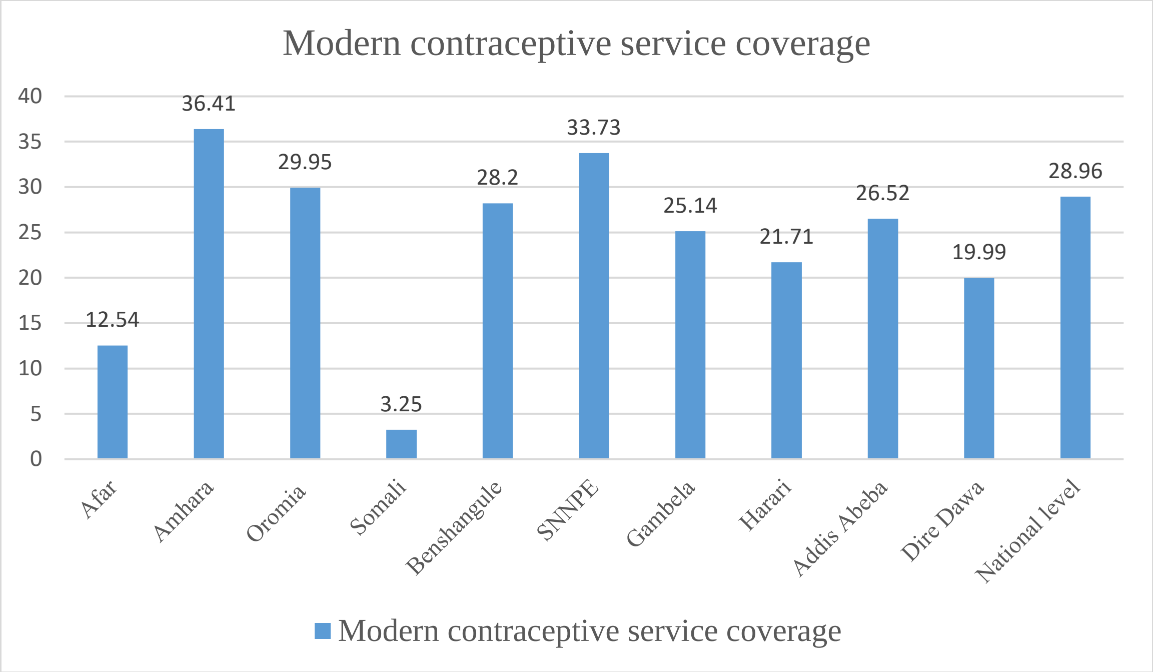

Figure 1: Crude coverage of modern contraceptive use in Ethiopia, 2019

Quality of modern contraceptive

The modern contraception service had an overall quality score of 66.84% (95% CI: 66.04, 67.63). The quality varies from Addis Ababa's 73.69% to Gambela's 58.81%. The outcome component had the highest quality score 90.24. The structural component had the second score (66.57%) ranging from 55.73% in Gambela to 77.53% in Addis Ababa. Lastly, the process component had 53.77% quality score, ranging from 47.95% in Afar to 60.68% in Addis Ababa (Table 3).

Table 3: Quality of family planning service in Ethiopia

Region	Facility	Structural (%)	Process (%)	Outcome (%)	Subtotal
		57.71	47.95	90.07	61.29
**Amhara**	117	71.51	53.38	92.50	68.92
**Oromia**	164	65.86	53.56	91.13	66.71
**Somali**	26	63.34	48.02	76.92	60.17
**Benshangule**	29	65.61	48.37	97.96	66.08
**SNNPE**	100	66.02	56.63	90.54	67.91
**Gambela**	51	55.73	48.73	81.08	58.81

**Harari**	11	66.50	60.53	92.51	70.17
**Addis Abeba**	43	77.53	60.68	90.97	73.69
**Dire Dawa**	20	71.75	54.58	94.11	69.89
**National level**	648	66.57	53.77	90.24	66.84

The quality of modern contraceptive varies with the management authority of Health facilities, NGOs had the highest quality (73.24%) with higher and private facilities score the lowest quality (58.11%). Moreover, According to areas of health facilities, facilities in urban areas had higher quality (68.54%). (Figure 2).

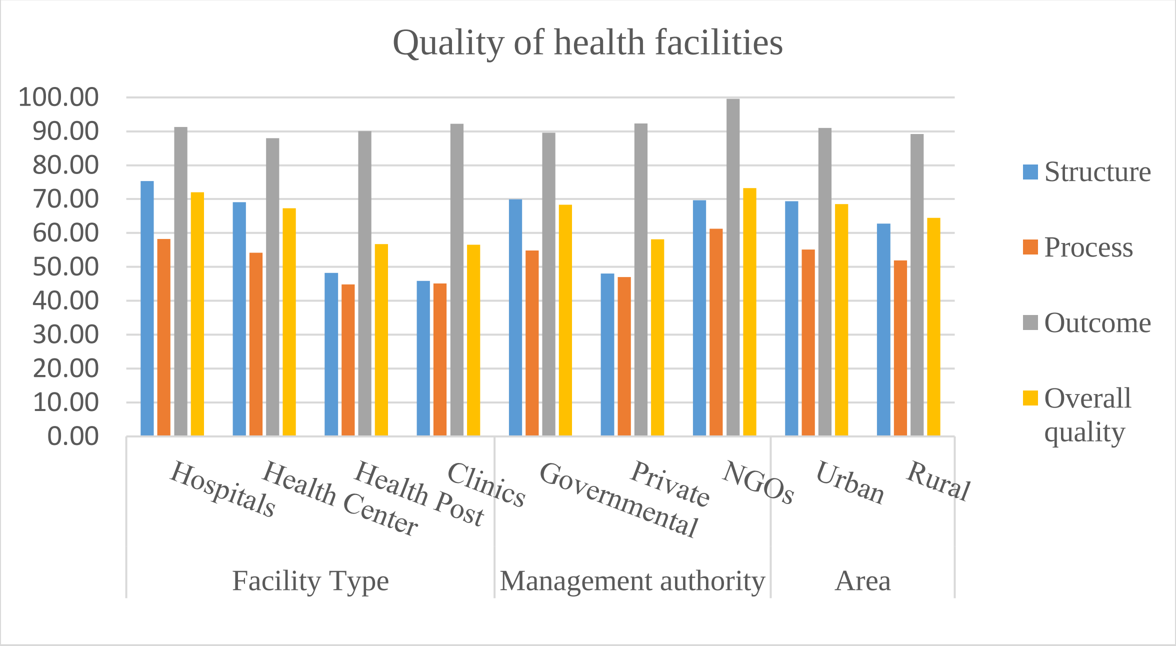

Figure 2: Quality score by facility characteristic in Ethiopia, 2019

The quality of modern contraceptive also varies with the type of health facilities, hospitals had the highest quality (71.98%) where hospitals in Harari had best quality 80.32% whereas hospitals in Gambela had lesser quality (63.64%) (Table 4).

Table 4: Quality of health facilities by regional Ethiopian SPA 2020-2021

**Region**	**Facility**	**Quality (%)**				
		**H**	**HC**	**HP**	**C**	**Subtotal**
**Afar**	32	66.97	63.43	48.60	59.63	61.29

**Amhara**	117	71.99	69.71	56.68	58.18	68.92
**Oromia**	164	71.92	65.12	56.53	54.31	66.71
**Somali**	26	73.02	54.32	50.69	52.85	60.17
**Benshangule**	29	78.46	69.47	58.33	53.00	66.08
**SNNPE**	100	71.12	66.89	61.37	60.19	67.91
**Gambela**	51	63.64	64.62	52.02	55.09	58.81
Harari	11	80.32	72.10	56.78	N/A	70.17
**Addis Abeba**	43	76.58	73.98	N/A	53.39	73.69
**Dire Dawa**	20	69.28	71.98	56.24	71.21	69.89
**National level**	648	71.98	67.35	56.71	56.54	66.84

SNNPE: Southern Nations, Nationalities and Peoples

Effective coverage

The national effective coverage of modern contraceptive service was 19.51% (95% CI: 19.03, 19.99) with a large variation in different regions, ranging from 7.69% in Afar to 25.09% in Amhara. Amhara had the highest effective coverage of modern contraceptive service 25.09% with a 11.32% difference from its crude coverage (36.41%) (Table 5).

Table 5: Effective coverage of modern contraceptive service in Ethiopia

**Region**	**Crude coverage (%)**	**EC by region**	**CC-EC (% point)**
Afar	12.54	7.69	4.85
Amhara	36.41	25.09	11.32
Oromia	29.95	19.98	9.97
Somali	3.25	1.96	1.29
Benshangule	28.20	18.64	9.56
SNNPE	33.73	22.91	10.82
Gambela	25.14	14.79	10.35
Harari	21.71	15.23	6.48
Addis Abeba	26.52	19.55	6.97
Dire Dawa	19.99	13.97	6.02
National level	28.96	19.51	9.45

SNNPE: Southern Nations, Nationalities and Peoples

Spatial distribution and hotspot analysis of Effective coverage of modern contraceptive

Effective modern contraceptive coverage showed a significant positive spatial autocorrelation in Ethiopia with the Global Moran's index value of 0.56 with (p< 0.001) (Figure 3).

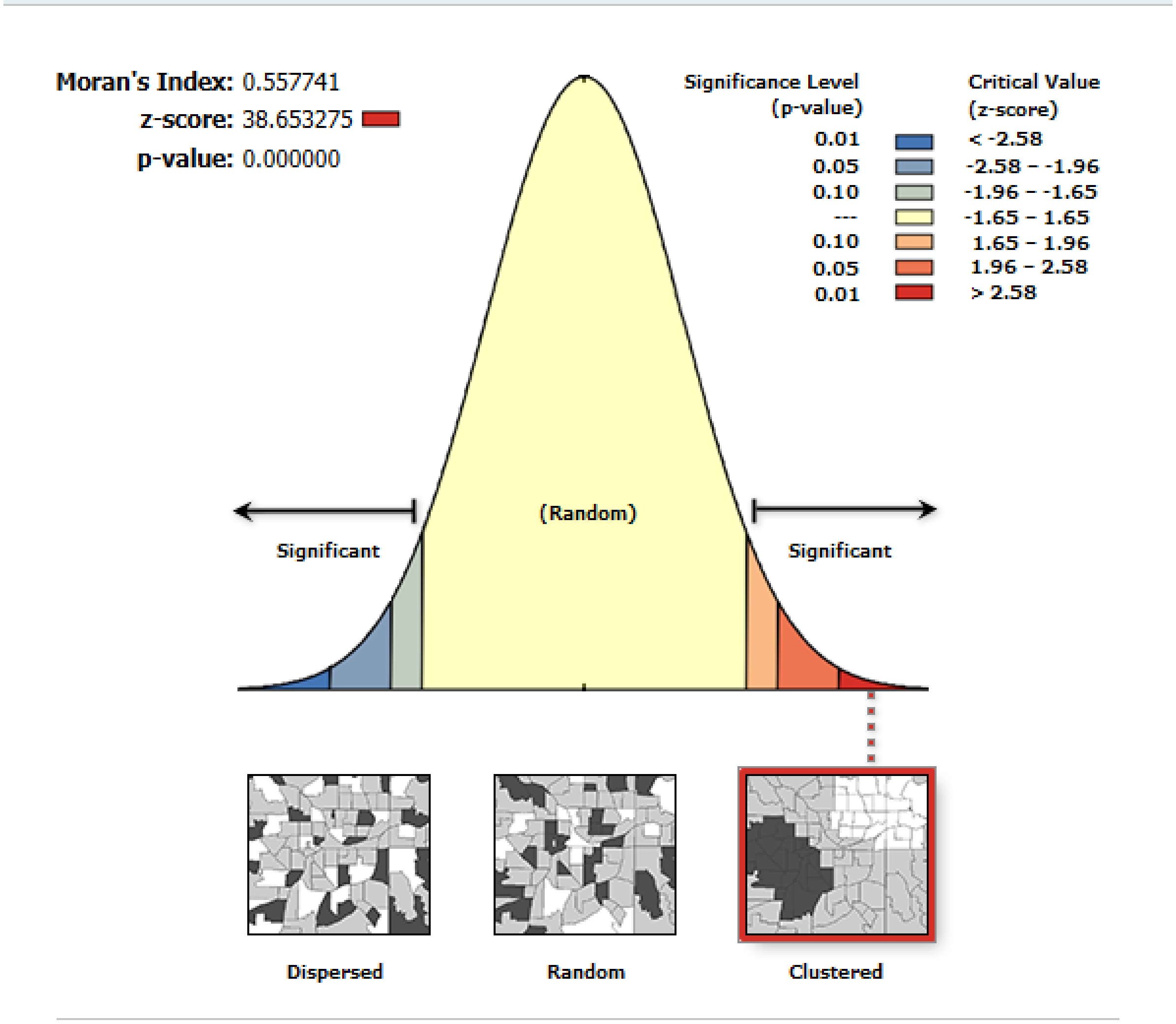

Figure 3: Spatial autocorrelation analysis of effective coverage of modern contraceptive

The spatial distribution analysis showed that facilities found in Amhara, Addis Ababa and the Northern part of SNNPE had higher effective coverage (Figure 4).

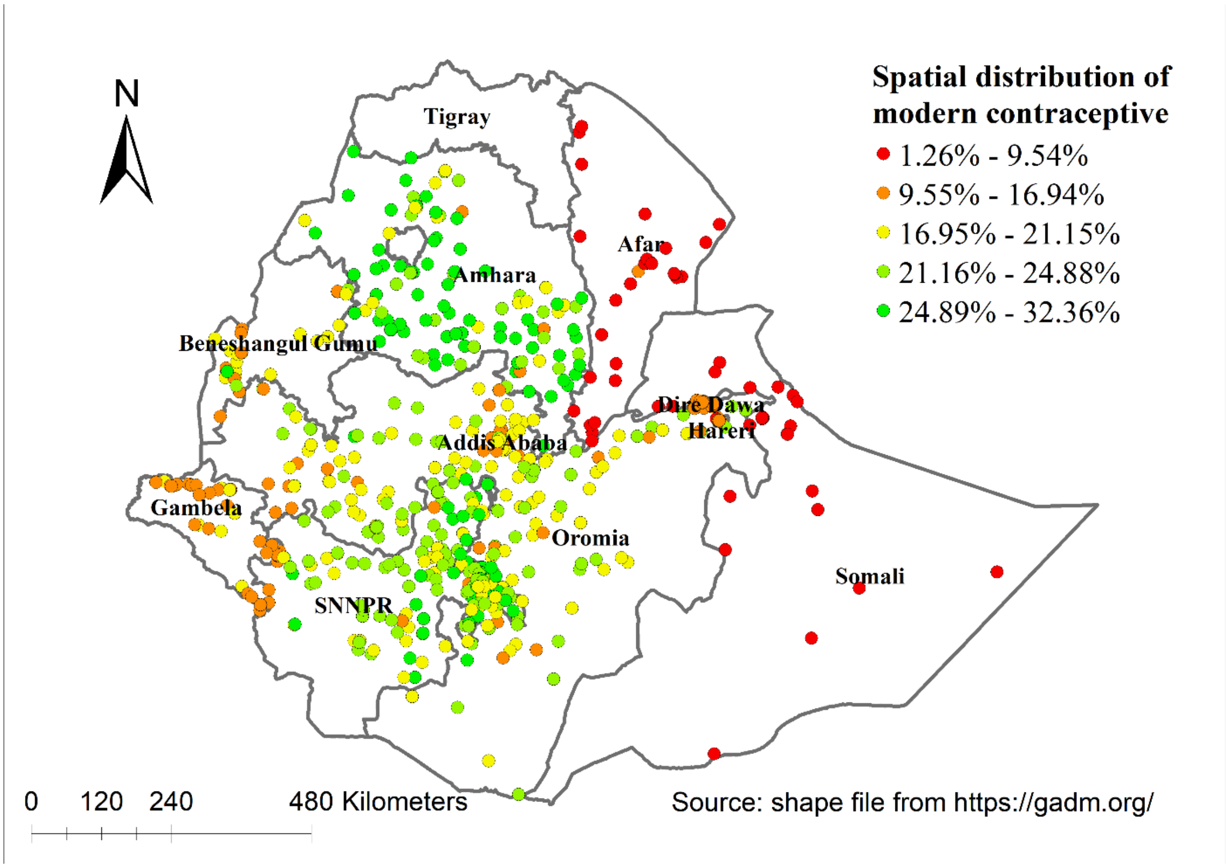

Figure 4: Spatial distribution of effective coverage of modern contraceptive in Ethiopia

There were hot and cold spots areas for effective coverage of modern contraceptive in Ethiopia. Addis Ababa, Amhara, North SNNP, and Northern Oromia had hotspots for effective coverage of modern contraceptive. On the other hand, Afar, Gambela, Dire Dawa, Harrarie and Somali had cold spots for Effective coverage of modern contraceptive (Figure 5).

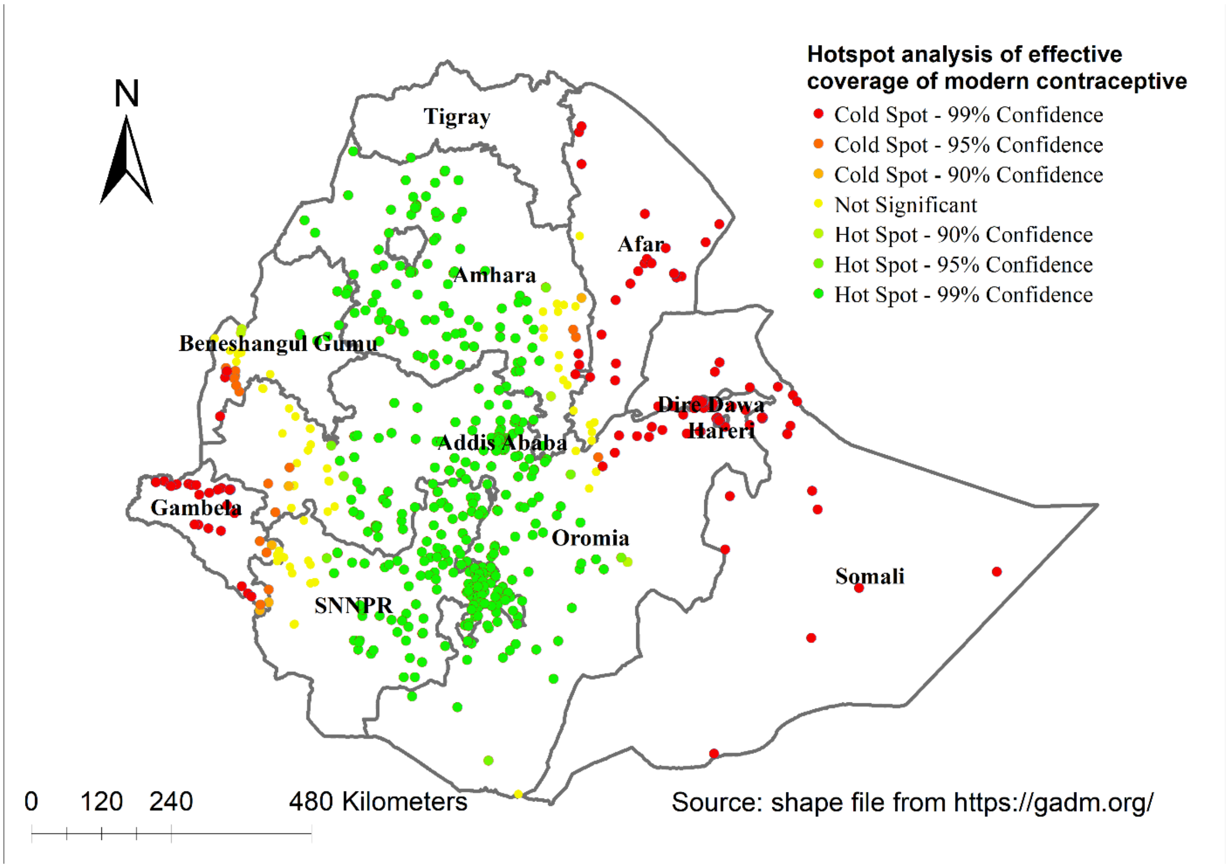

Figure 5:Hotspot analysis of effective coverage of modern contraceptive service in Ethiopia

Spatial interpolation of modern contraceptive in Ethiopia

The spatial prediction of effective coverage of modern contraceptive showed a high coverage mainly in Addis Ababa, Amhara and Dire Dawa. There were low Effective coverage of modern contraceptive in Somali, Afar, and Gambela. The predicted low Effective coverage of modern contraceptive was shown in red color (Figure 6).

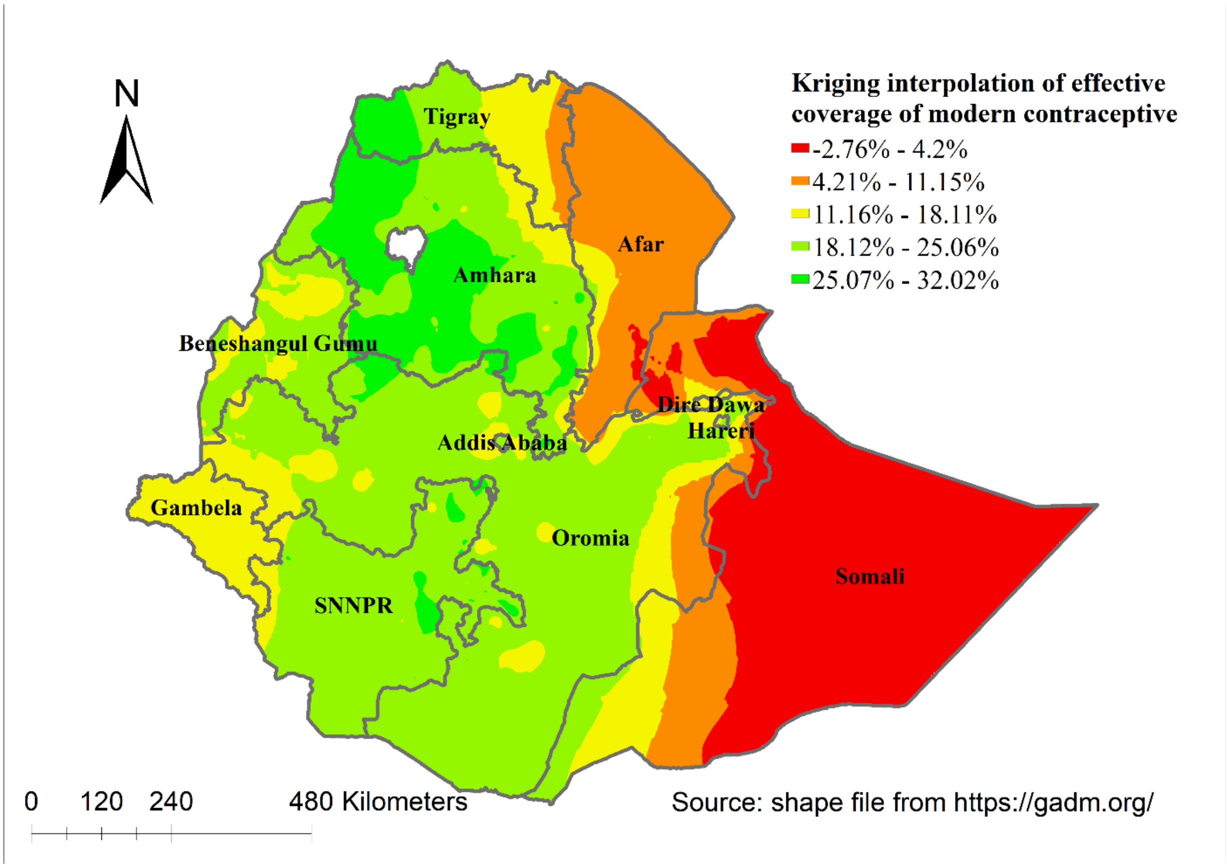

Figure 6:Spatial interpolation of effective coverage of modern contraceptive service in Ethiopia

Discussion

This study revealed the effective coverage and quality of modern contraceptive service in Ethiopia using the three components of quality measurements. The national effective coverage of modern contraceptive service was 19.51% with a large variation in different regions, ranging from 7.69% in Afar to 25.09% in Amhara. Previous similar study in Ethiopia also found a low effective coverage of family planning service with a large variation in geographical area of health facilities [14, 29]. A study conducted in different African countries reported that the effective coverage of primary health service were very low [30]. This might be due to low quality of health facility infrastructure, health service supplies and experienced health provider. Another reason might be, availability of health service and accessibility of health service in peripheral regions which results in missing the opportunity to give quality family planning.

Improved quality of family planning service will increase the service utilization of women [31], which as a result increase the effective coverage of family planning service. The modern contraception service had an overall quality score of 66.84%. Supporting this a study conducted in Ethiopia had reported low quality of family planning service [32]. The quality varies from Addis Ababa's 73.69% to Gambela's 58.81%. A study conducted on high quality on family counseling reported that the quality differ in health facilities geographical location [33].Moreover, Facilities in urban areas had higher quality (68.54%). A study conducted in Ethiopia founds out that clients in urban facilities were more likely to had quality service than clients in rural [34]. A study conducted in Lao PDR stated that, there was a significant quality difference in family planning service in urban and rural facilities[35]. This might be due to experienced personals were more likely to employed in urbanized area. Another possible reason might be low infrastructure and the distance is challenges to take health supplies to peripheral area.

The quality of modern contraceptive varies with the management authority of Health facilities, NGOs had the highest quality (73.24%) with higher and private facilities score the lowest quality (58.11%). This reported was evidenced with previous study conducted in Ethiopia comparing private and public facilities on family planning service, and stated that private facilities had shortage of availability of supplies, FP guidelines, trained providers, and quality assurance activities [36]. This might be due to NGOs had financial resources that allow them to fulfill family planning service equipment and employ highly qualified health professionals.

The study use women satisfaction to measure the quality of outcome components. Outcome component had the highest quality score 90.24%. This was consistent with a study in Jimma zone, Southwest Ethiopia (93.7%)[37], The finding was higher than a study conducted in Mozambique (85%) [38], Nigeria (81%) [39]. The structural component had the second score (66.57%) ranging from 55.73% in Gambela to 77.53% in Addis Ababa. A study conducted in Ethiopia had showed that there was low availability of supplies and different infrastructure of family planning service in privates facilities which intern decrees the overall quality[36]. Lastly, the process component had 53.77% quality score, ranging from 47.95% in Afar to 60.68% in Addis Ababa. A study conducted in Ethioipia reported a low quality of family planning counseling service [33]. Accordingly, there was a need to monitor and improve each quality components to had the desired service quality.

Strength and limitation of the study

This study had numerous strengths, including the use of the DHS dataset, which had a high sample size, as well as the most recent EDHS dataset 2019. However, the study had some limitations, The first limitation of the study was that we did not exclude the use of contraceptives from other sources, such as pharmacies, because EDHS 2019 did not contain any observations regarding the source of contraceptives. The other limitation was that the Tigray region was not included as the SPA does not had a record for facilities in Tigray and only facilities which had records for exit interview were included.

Conclusion

The study found that, Ethiopia had a poor effective coverage of modern contraceptives services. Somalia and Afar regions had less effective coverage of modern contraceptive service. The study found that, Ethiopia had a poor effective coverage of modern contraceptives services. Somalia and Afar regions had less effective coverage of modern contraceptive service. Addis Ababa, Amhara, North SNNP, and Northern Oromia had hotspots for effective coverage of modern contraceptive. On the other hand, Afar, Gambela, Dire Dawa, Harrarie and Somali had cold spots for Effective coverage of modern contraceptive. Policymakers and responsible bodies should focus on improving the quality of the facilities in rural areas and the facilities in Gambela, Somali and Afar. Policymakers and responsible bodies should focus on improving the quality of the facilities in rural areas and the facilities in Gambela, Somali and Afar.

Declarations

Ethics approval and consent to participate

The Ethiopian demographic health survey (DHS) dataset is available to the general public upon request from the Measure DHS website http://www.measuredhs.com. We submitted a request to the DHS by briefly stating the objectives of this analysis and thereafter received permission to download the maternal record dataset.

Patient consent for publication: Not applicable

Availability of data and materials

The dataset is available on the Measure DHS website: http://www.measuredhs.com.

Competing interests

The authors declare that they have no competing interests.

Funding

Not applicable

Authors' contributions

SBA: Conceptualization, Methodology, Formal analysis, Writing – Original Draft, Visualization.

MYB: Methodology, Formal Analysis, Writing – Original Draft

MTY: Methodology, Formal Analysis, Writing – Original Draft

MBA: Conceptualization, Methodology, Formal analysis, Manuscript preparation,

Reference

1. Ayele W, Tesfaye H: **Trends and determinants of unmet need for family planning and programme options, Ethiopia: Further analysis of the 2000, 2005, and 2011 Demographic and Health Surveys**: ICF International; 2013.
2. Festin MPR, Kiarie J, Solo J, Spieler J, Malarcher S, Van Look PF, Temmerman M: **Moving towards the goals of FP2020—classifying contraceptives**. *Contraception* 2016, **94**(4):289-294.
3. Marquez MP, Kabamalan MM, Laguna E: **Traditional and modern contraceptive method use in the Philippines: trends and determinants 2003–2013**. *Studies in Family Planning* 2018, **49**(2):95-113.
4. Cahill N, Sonneveldt E, Stover J, Weinberger M, Williamson J, Wei C, Brown W, Alkema L: **Modern contraceptive use, unmet need, and demand satisfied among women of reproductive age who are married or in a union in the focus countries of the Family Planning 2020 initiative: a systematic analysis using the Family Planning Estimation Tool**. *The Lancet* 2018, **391**(10123):870-882.
5. **Family planning/contraception methods** [https://www.who.int/news-room/fact-sheets/detail/family-planning-contraception]
6. Gebre MN, Edossa ZK: **Modern contraceptive utilization and associated factors among reproductive-age women in Ethiopia: evidence from 2016 Ethiopia demographic and health survey**. *BMC Women's Health* 2020, **20**(1):1-14.
7. Organization WH: **World health statistics 2016: monitoring health for the SDGs sustainable development goals**: World Health Organization; 2016.

8. Liu L, Oza S, Hogan D, Perin J, Rudan I, Lawn JE, Cousens S, Mathers C, Black RE: **Global, regional, and national causes of child mortality in 2000–13, with projections to inform post-2015 priorities: an updated systematic analysis**. *The lancet* 2015, **385**(9966):430-440.
9. Bitew F, Nyarko SH: **Modern contraceptive use and intention to use: implication for under-five mortality in Ethiopia**. *Heliyon* 2019, **5**(8):e02295.
10. Cleland J, Bernstein S, Ezeh A, Faundes A, Glasier A, Innis J: **Family planning: the unfinished agenda**. *The lancet* 2006, **368**(9549):1810-1827.
11. Kruk ME, Gage AD, Arsenault C, Jordan K, Leslie HH, Roder-DeWan S, Adeyi O, Barker P, Daelmans B, Doubova SV: **High-quality health systems in the Sustainable Development Goals era: time for a revolution**. *The Lancet global health* 2018, **6**(11):e1196-e1252.
12. Donabedian A: **The quality of care: how can it be assessed?** *Jama* 1988, **260**(12):1743-1748.
13. Tanahashi T: **Health service coverage and its evaluation**. *Bulletin of the World Health organization* 1978, **56**(2):295.
14. Yakob B, Gage A, Nigatu TG, Hurlburt S, Hagos S, Dinsa G, Bowser D, Berman P, Kruk ME, Tekle E: **Low effective coverage of family planning and antenatal care services in Ethiopia**. *International Journal for Quality in Health Care* 2019, **31**(10):725-732.
15. Ng M, Fullman N, Dieleman JL, Flaxman AD, Murray CJ, Lim SS: **Effective coverage: a metric for monitoring universal health coverage**. *PLoS medicine* 2014, **11**(9):e1001730.
16. Shengelia B, Tandon A, Adams OB, Murray CJ: **Access, utilization, quality, and effective coverage: an integrated conceptual framework and measurement strategy**. *Social science & medicine* 2005, **61**(1):97-109.
17. Tegegne TK, Chojenta C, Forder PM, Getachew T, Smith R, Loxton D: **Spatial variations and associated factors of modern contraceptive use in Ethiopia: a spatial and multilevel analysis**. *BMJ open* 2020, **10**(10):e037532.
18. Alemayehu GA, Fekadu A, Yitayal M, Kebede Y, Abebe SM, Ayele TA, Gizaw Z, Wubeshet M, Muchie KF, Gelagay AA: **Prevalence and determinants of contraceptive utilization among married women at Dabat Health and Demographic Surveillance System site, northwest Ethiopia**. *BMC women's health* 2018, **18**:1-7.
19. Belete N, Zemene A, Hagos H, Yekoye A: **Prevalence and factors associated with modern contraceptive discontinuation among reproductive age group women, a community based cross-sectional study in Humera town, northern Ethiopia**. *BMC women's health* 2018, **18**:1-8.
20. Worku AG, Tessema GA, Zeleke AA: **Trends of modern contraceptive use among young married women based on the 2000, 2005, and 2011 Ethiopian demographic and health surveys: a multivariate decomposition analysis**. *PloS one* 2015, **10**(1):e0116525.
21. Ministry of Health FDRoE: **National Guideline for Family Planning Services in Ethiopia**. In.: Ethiopia: FMOH Addis Ababa; 2020.
22. Haile TG, Asefa A, Mirkuzie A, Benova L: **Effective coverage of curative child health services in Ethiopia? tracking progress towards universal health coverage**. In: *33rd EPHA Annual Conference: 2022*; 2022.
23. **A Comparison of Summary Measures of Quality of Service and Quality of Care for Family Planning in Haiti, Malawi, and Tanzania** [https://dhsprogram.com/pubs/pdf/MR20/MR20.pdf]
24. Marsh AD, Muzigaba M, Diaz T, Requejo J, Jackson D, Chou D, Cresswell JA, Guthold R, Moran AC, Strong KL: **Effective coverage measurement in maternal, newborn, child, and adolescent health and nutrition: progress, future prospects, and implications for quality health systems**. *The Lancet Global Health* 2020, **8**(5):e730-e736.
25. McMillen DP: **Geographically weighted regression: the analysis of spatially varying relationships**. In.: JSTOR; 2004.

26. Belay DG, Asratie MH, Gashaw M, Tsega NT, Endalew M, Aragaw FM: **Community and individual level determinants and spatial distribution of deworming among preschool age children in Ethiopia: spatial and multi-level analysis**. *BMC Public Health* 2022, **22**(1):872.
27. Mitas L, Mitasova H: **Spatial interpolation**. *Geographical information systems: principles, techniques, management and applications* 1999, **1**(2):481-492.
28. Oliver MA, Webster R: **Kriging: a method of interpolation for geographical information systems**. *International Journal of Geographical Information System* 1990, **4**(3):313-332.
29. Getnet M, Gebremedhin S, Melesse DY, Munos MK, Hazel EA, Wado YD, Zeru A, Worku A: **Effective Coverage of Modern Contraceptive Use in Ethiopia: An Ecological Linking Analysis of Service Provision Assessment and National Health Equity Surveys**. *International Journal of Environmental Research and Public Health* 2024, **21**(12):1570.
30. Leslie HH, Ndiaye Y, Kruk ME: **Effective coverage of primary care services in eight high-mortality countries**. *BMJ global health* 2017, **2**(3):e000424.
31. Nurjaeni N, Sawangdee Y, Pattaravanich U, Holumyong C, Chamratrithirong A: **The role of structural and process quality of family planning Care in Modern Contraceptive use in Indonesia: a multilevel analysis**. *BMC Public Health* 2021, **21**:1-15.
32. Fruhauf T, Zimmerman L, Kibira SPS, Makumbi F, Gichangi P, Shiferaw S, Seme A, Guiella G, Tsui A: **Measuring family planning quality and its link with contraceptive use in public facilities in Burkina Faso, Ethiopia, Kenya and Uganda**. *Health Policy and Planning* 2018, **33**(7):828-839.
33. Ejigu BA, Seme A, Zimmerman L, Shiferaw S: **Trend and determinants of quality of family planning counseling in Ethiopia: Evidence from repeated PMA cross-sectional surveys,(2014–2019)**. *PloS one* 2022, **17**(5):e0267944.
34. Tessema GA, Mahmood MA, Gomersall JS, Assefa Y, Zemedu TG, Kifle M, Laurence CO: **Client and facility level determinants of quality of care in family planning services in Ethiopia: Multilevel modelling**. *PLoS One* 2017, **12**(6):e0179167.
35. Thongmixay S, Schoneveld T, Vongxay V, Broerse JEW, Sychareun V, Essink DR: **Quality of family planning services for women of reproductive age in Lao PDR**. *Global Health Action* 2020, **13**(sup2):1788261.
36. Tessema GA, Mahmood MA, Gomersall JS, Assefa Y, Zemedu TG, Kifle M, Laurence CO: **Structural quality of services and use of family planning services in primary health care facilities in Ethiopia. How do public and private facilities compare?** *International journal of environmental research and public health* 2020, **17**(12):4201.
37. Tafese F, Woldie M, Megerssa B: **Quality of family planning services in primary health centers of Jimma Zone, Southwest Ethiopia**. *Ethiopian journal of health sciences* 2013, **23**(3):245-254.
38. Galle A, Vermandere H, Griffin S, de Melo M, Machaieie L, Van Braeckel D, Degomme O: **Quality of care in family planning services in rural Mozambique with a focus on long acting reversible contraceptives: a cross-sectional survey**. *BMC women's health* 2018, **18**:1-13.
39. Kaoje UA, Sambo M, Oche M, Saad A, Raji M, Isah B: **Determinants of client satisfaction with family planning services in government health facilities in Sokoto, Northern Nigeria**. *Sahel Medical Journal* 2015, **18**(1):20.

Click here to access/download
Supporting Information
Supplementary File.docx
