# Supplementary material for: Mapping the effective coverage of modern contraceptive services in Ethiopia
Source: PLoS One. 2025 Jul 7;20(7):e0327581. doi: 10.1371/journal.pone.0327581 (PMC12233249; doi:10.1371/journal.pone.0327581)
Supplement: Attachment — Submitted filename: Comment as a reviwer for EDHS data.docx [file pone.0327581.s003.docx]

**Abstract**

- Avoid using acronyms at this section
- Please include the clear gap beyond the coverage.
- It would be better if you included keywords

**Introduction**

- There is an idea stated “Therefore, this indicates the prevalence of unmet need for modern contraceptives among married or in-union women is around 10% [7].” in paragraph 2.
- It is not clear how the unmet need was calculated
- And even it is better to focus on the study area like sub-Saharan, Ethiopia
- Try to include the figures how the poor quality affects the quality of health.
- Try to include how poor quality related with effective coverage.
- Despite it was not adjusted with quality of service, I recommend you to include the figure of the coverage.
- There are acronyms without any elaborations in their first stand

**Methods**

1. Measurement

- How did you measure the frequency and or duration of contraceptive use? Is it a history/ever used or a current use?
- How the quality of measurement was determined? When did you say poor quality or good quality?

2. There are missed components that had to be reported despite it was a secondary data analysis.

- What was the study design?
- What was the study population?
- What was the sampling technique?
- How did you assure the data quality?
- Ethical consideration

3. Data analysis

- Spatial autocorrelation, spatial interpolation, and hotspot analysis had to be written separately as subheadings.
- What model was used? How the model chosen?
- How the samples weighted?
- What way of measurement was used to measure variations between regions?

**Result**

- Your text report regarding “Age” is inappropriate. It says 42.25% below 42 and 21.74% above 36: What does it mean? Does it mean 0-24 and 36 to ……….?
- The total number of participants (n) is not the same as that you stated in some variables. For example marital status (n=7619).
- Why this happened?
- Better to include “total” as a category for each variable
- Categories of some variables are not logical sequenced. For example region
- I recommend you to sequence variables in logical manner using their frequency either ascending or descending
- Some graphs lacks the minimum standard that should contain like Legend, caption
- The footnote for table 5 is not written in a recommended way.
- Better to state it under the table in text form with a specific note (to attract the reader)

**Discussion**

- In some points you tried to discuss other’s study rather than yours which is not a recommended scientific way of writing.
- In some points you said various studies/many studies/different studies but you cited only a single reference.
- In some points you said other’s finding low coverage and or quality.
- Why preferred to report or compare and contrast in this way?
- Why didn’t you compare and contrast in a quantitative way (or why didn’t you show their difference and or similarity rather than concluding others?) which is measurable to judge for the readers.
- It even difficult to conclude other’s finding
- You discussed with a study done on counseling quality which is not similar with yours.
- There is an idea which says “Moreover, Facilities in urban areas had higher quality (68.54%).”
- Is it your finding or others’?
- Whether it is your or others’, it is not a recommended scientific ways of writing.

**Conclusion**

- “The study found that, Ethiopia had a poor effective coverage of modern contraceptives services”
- This idea is a copy paste of the first sentence.
- “Somalia and Afar regions had less effective coverage of modern contraceptive service.”
- This is also repeated
- “Policymakers and responsible bodies should focus on improving the quality of the facilities in rural areas and the facilities in Gambela, Somali and Afar”
- This is also repeated
