# Supplementary material for: Mapping the effective coverage of modern contraceptive services in Ethiopia
Source: PLoS One. 2025 Jul 7;20(7):e0327581. doi: 10.1371/journal.pone.0327581 (PMC12233249; doi:10.1371/journal.pone.0327581)
Supplement: Attachment — Submitted filename: A point by point response.docx [file pone.0327581.s007.docx]

**Point by point responses**

We sincerely appreciate the valuable feedback and constructive comments provided by the editor and reviewers. We have carefully considered all the suggestions and have thoroughly revised the manuscript to enhance its clarity and methodological rigor.

**Reviewer 1**

**Abstract**

Q1: Avoid using acronyms at this section

R1: Thank you for your feedback. We have revised it to avoid using acronyms accordingly.

*“We used the 2019 Ethiopian Demographic Health Survey (EMDHS) to estimate the coverage of modern contraceptive services. In addition, we used the 2021-2022 Ethiopian Service Provision Assessment (ESPA) to estimate the quality of modern contraceptive service provision The two datasets were linked using Euclidean method. The effective coverage was calculated as the product of coverage and quality estimates for each health facility. The geospatial estimates were presented using the coordinates from the ESPA survey. We analyzed the spatial distribution of modern contraceptive coverage using ArcGIS 10.7 software. The Global Moran's I statistic was used to assess the spatial autocorrelation, while the Getis-Ord Gi* statistic was used to identify high and low clusters. The Kriging interpolation method was used to estimate effective coverage in unsampled areas based on sampled clusters.” (Page 2 line 5-9)*

Q2: Please include the clear gap beyond the coverage.

R2: Thank you for your valuable feedback. We have revised the manuscript.

*“The national effective coverage of modern contraceptive services was 20% (95% CI: 19.87, 21.82), which is notably lower than the crude coverage of 30% (95% CI: 28.93, 31.62), resulting in a gap of 10% points. The spatial distribution analysis showed that effective modern contraceptive coverage had a significant positive spatial autocorrelation in Ethiopia with the Global Moran's index value of 1.92 with (p< 0.001). The facilities found in central Amhara, western Amhara, central Oromia, western part of Southern Nations, Nationalities and Peoples (SNNP), eastern SNNP and the northern part of SNNP had higher effective coverage. Addis Ababa, western Amhara, northeast Benishangul-Gumuz, north SNNP, eastern SNNP and northern and central Oromia had hotspots for effective coverage of modern contraceptive. On the other hand, Afar, western Gambela, western Oromia, western Benishangul-Gumuz, Dire Dawa, Harari and Somali had cold spots for effective coverage of modern contraceptive.” (Page 2 line 17-26)*

Q3: It would be better if you included keywords

R3: Thank you for your suggestion. We have now included the following

*“Keywords: Effective coverage, Modern contraceptive, Quality-adjusted coverage, Spatial distribution, Inequality, Ethiopia “(Page 3 line 10)*

**Introduction**

Q4: There is an idea stated “Therefore, this indicates the prevalence of unmet need for modern contraceptives among married or in-union women is around 10% [7].” in paragraph 2. It is not clear how the unmet need was calculated

R4: We appreciate your valuable input. We have revised the statement.

*“Globally, 77% of the 1.2 billion women of reproductive age (15–49) needing family planning were using modern contraceptives in 2022, leaving approximately 270 million women with unmet need (8). Regional inequalities persist, with Sub-Saharan Africa bearing the greatest burden, modern contraception remains inaccessible to 37% of women in the region (8). According to the 2019 Ethiopian Demographic and Health Survey (EDHS), the national contraceptive prevalence for modern methods is around 28.1%, with significant regional variation. In Addis Ababa, coverage is higher at 47.6%, while in Somali, it is much lower, at about 3.4% (9).” (Page 4 line 15-21)*

Q5: And even it is better to focus on the study area like sub-Saharan, Ethiopia. Try to include the figures how the poor quality affects the quality of health. Try to include how poor quality related with effective coverage. Despite it was not adjusted with quality of service, I recommend you to include the figure of the coverage. There are acronyms without any elaborations in their first stand

R5: Thank you for your valuable feedback. We have revised the manuscript by including figures that demonstrate how poor-quality affects health outcomes and how poor quality is related to effective coverage. Additionally, we have added figures on coverage and ensured that acronyms are properly elaborated when they first appear in the text to improve clarity and comprehension. *(Page 5 line 3-19)*

**Methods**

1.	**Measurement**

Q6: How did you measure the frequency and or duration of contraceptive use? Is it a history/ever used or a current use?

R6: Thank you for your comment. The coverage measures in this study were derived from the current use of modern contraceptives, ensuring that the analysis reflects the most recent and relevant data on contraceptive utilization. We have clarified this in the manuscript for better understanding. *(Page 9 line 3)*

Q7: How the quality of measurement was determined? When did you say poor quality or good quality?

R7: Thank you for your comment. In this study, we did not classify quality as poor or good. Instead, we used a quality score, which was then multiplied by the coverage to assess quality-adjusted coverage. We have clarified this in the manuscript to avoid any misunderstanding.

Q8:	There are missed components that had to be reported despite it was a secondary data analysis.

R8: Thank you for your valuable feedback. We have now incorporated detailed explanations for each of these components in the Methods section, including the study design, study population, sampling technique, data quality assurance, and ethical considerations to ensure clarity and completeness.

Q8.1: What was the study design?

R8.1*: “This study used a cross-sectional design based on secondary data from the Ethiopian Service Provision Assessment and the Ethiopian Demographic and Health Survey. The 2019 mini-EDHS data, which was intended to offer estimates of important indicators for the nation as a whole, for urban and rural areas separately, for each of the nine regions, and the two administrative cities, and SPA 2020–2021, which is the second facility assessment that gathers information on service availability and quality of care, was used in this study. The EDHS holds publicly available data at Measure DHS at https://dhsprogram.com.”* *(Page 6 line 11-18)*

Q8.2: What was the study population?

R9.2: We have also included figure to illustrate this sampling technique.

*“To assess contraceptive coverage, we analyzed data from the individual record (IR) file of the Ethiopia Demographic and Health Survey (EDHS). Our initial sample consisted of 8,885 women of reproductive age at the time of the survey. However, we excluded 722 pregnant women from the dataset to ensure the accuracy of contraceptive coverage estimates. Furthermore, we removed data from the Tigray region for consistency in data analysis, as no corresponding records are available in the Ethiopia Service Provision Assessment (ESPA) dataset. This resulted in excluding 733 observations from Tigray, leaving a final unweighted sample of 7,430 women.*

*To measure the quality of contraceptive services, we utilized health facility and family planning records from the ESPA dataset, which contains information from 1,158 health facilities nationwide. For our study, we specifically selected 648 health facilities that had available exit interview records related to family planning services.” (Page 6 line 11-18)*

Q8.3: What was the sampling technique?

R9.3: *“The 2019 Ethiopian Mini Demographic and Health Survey (EMDHS) used the 2019 Ethiopian Population and Housing Census as its sampling frame, which included 149,093 enumeration areas (EAs), each averaging 131 households. The survey followed a two-stage stratified sampling approach, selecting 305 EAs proportionally across urban and rural areas. A household listing was conducted, and 30 households per cluster were systematically chosen. Women aged 15–49 were interviewed, and height and weight measurements were collected from children under five. 8,885 of the 9,012 women who were eligible for individual interviews in the interviewed households finished the interview (9).*

*The 2021–22 Ethiopian Service Provision Assessment used a stratified random sample of 1,407 health facilities. All 413 hospitals were included due to their crucial role in the health system. A representative sample of 310 health centres, 328 health posts, and 356 clinics was selected, with oversampling of key facilities to improve survey precision. Data collection covered 1,158 facilities (82% of the sample), with some exclusions due to closures, security concerns, or facility conversions. Most facilities were health posts (65%), primarily government-managed (83%), and located in rural areas (77%), with the highest concentration in Oromia, Amhara, and SNNPR regions (29).” (Page 6 line 19-27)*

Q8.4: How did you assure the data quality?

R9.4: *“Based on the standard Demographic and Health Survey questionnaires used by the DHS Program, which were modified to account for the Ethiopian population and health concerns, the DHS health survey employs a carefully crafted questionnaire. Before the actual survey, the data collection staff received training and pretesting. Furthermore, quality controllers provided assistance and oversight during the data collection process (24). Fieldwork supervision was conducted by EPHI, MoH, and ICF personnel, with regional coordinators monitoring data collection. Automated quality checks, field check tables, and double data entry were used to maintain data accuracy.” (Page 7 line 10-16)*

Q9.5: Ethical consideration

R9.5: *“The Ethiopian demographic health survey (DHS) dataset is available to the general public upon request from the DHS website https://dhsprogram.com/. We submitted a request to the DHS by briefly stating the objectives of this analysis and thereafter received permission to download the maternal record dataset.*

*Patient and public involvement: Patients and/or the public were not involved in the design, or conduct, or reporting, or dissemination plans of this research.” (Page 11 line 24)*

3.	**Data analysis**

Q10: Spatial autocorrelation, spatial interpolation, and hotspot analysis had to be written separately as subheadings. What model was used? How the model chosen? How the samples weighted? What way of measurement was used to measure variations between regions?

R10: Thank you for your insightful feedback. We have now separated Spatial Autocorrelation, Spatial Interpolation, and Hotspot Analysis into distinct subheadings for better clarity. Additionally, we have provided details. We have also included effective coverage result with administrative linkage to acknowledge methodological difference. *(Page 10-11)*

*“The EDHS and ESPA datasets were linked using Euclidean (GPS-based) methods (35). The linkage was conducted in STATA using the "geonear" command, which matched health facilities to the closest EDHS survey cluster within a 15 km radius. If no cluster was found within this range, the health facility was linked to the nearest available cluster, even if it exceeded 15 km. In total, 244 DHS clusters and 648 health facilities were successfully linked. After linking the datasets, the effective coverage of modern contraceptive use was calculated by multiplying the quality of the health facility by the proportion of women using modern contraceptives in each cluster (26, 36). Finally, the average effective coverage was calculated for each health facility to map the effective coverage.*

*Effective coverage = Quality of the health service * crude coverage*

*The facility's characteristics, as well as the respondents' socioeconomic and demographic characteristics, were described using descriptive statistics. Lastly, tables and graphs were used to present the results. To account for methodological differences, an additional linkage approach was employed. Alongside the Euclidean (GPS-based) linkage, results were also reported using administrative linkage to provide a more comprehensive analysis (Supplementary file 2).*

***Spatial analysis***

*We use ArcGIS 10.7 software to analyse the spatial distribution after determining the effective coverage of modern contraceptives. The geospatial estimates were presented using the coordinates from the ESPA survey. Sampling weights were applied using sample weights from DHS to adjust for population distribution and ensure representativeness.*

***Spatial autocorrelation analysis***

*A measure of spatial autocorrelation called the Global Moran's I statistic was used to assess the spatial distribution of modern contraceptives that work in Ethiopia. The distribution of modern contraceptives across regions can be evaluated using this method to determine if it follows a spatial pattern or is random. Values close to −1 indicate dispersion, while values close to +1 indicate clustering. A Moran’s I value of zero suggests a random distribution. A statistically significant Moran’s I (p < 0.05) leads to rejection of the null hypothesis and indicates the presence of spatial autocorrelation (35).*

***Hots pot analysis (Getis-OrdGi* statistic)***

*A hot spot analysis was performed using the Getis-Ord Gi* statistic, which successfully identified noteworthy clusters of modern contraception that were high (hot places) and low (cold spots). To identify regions where modern contraceptive efforts are particularly successful or where coverage gaps are noticeable, this spatial statistical method is frequently used to find trends in geographic data. Z-scores and p-values were used to evaluate statistical significance, and 95% confidence intervals were used. A "hotspot" is denoted by a high Getis-Ord Gi* value, whereas a "cold spot" is denoted by a low Gi* value. Clustering's statistical significance was evaluated using Z-scores, and significance levels were evaluated using p-values. To depict spatial differences, low-value clusters (cold spots) and high-value clusters (hotspots) were mapped (36).*

***Spatial interpolation***

*The Kriging spatial interpolation approach was used to forecast the effective coverage of modern contraceptives in unsampled regions of Ethiopia based on sampled clusters (37). Kriging is a geostatistical technique that uses spatial autocorrelation and data from neighbouring sampled points to estimate values at unmeasured places. In areas where direct data collecting has not been carried out, this method guarantees more precise and trustworthy forecasts of the coverage of modern contraceptives (38).”*

**Result**

Q11: Your text report regarding “Age” is inappropriate. It says 42.25% below 42 and 21.74% above 36: What does it mean? Does it mean 0-24 and 36 to ……….?

R11: Thank you for your feedback. We have revised it as follows.

*“A total of weighted 7618 reproductive age women were included. Of these, the majority (42.25%) were aged 15-24 years, followed by 25-35 years (36.01%) and 36-49 years (21.74%). Over half (63.99%) were married, while 36.01% were unmarried. Most participants resided in rural areas (67.88%), while 32.12% were urban areas.” (Page 12 line 5-8)*

Q12: The total number of participants (n) is not the same as that you stated in some variables. For example, marital status (n=7619). Why this happened? Better to include “total” as a category for each variable

R12: Thank you for your observation. This was a typing error the correct number for unmarried women is 2,743, and we have corrected it. The total number of respondents is 7,618, which is correctly reported at the top of the table. Additionally, we have ensured consistency in the data presentation.

Q13: Categories of some variables are not logical sequenced. For example region. I recommend you to sequence variables in logical manner using their frequency either ascending or descending.

R13: Thank you for your suggestion. We have now rearranged the categories of variables, including regions, in a logical sequence based on their frequency.

Q14: Some graphs lack the minimum standard that should contain like Legend, caption. The footnote for table 5 is not written in a recommended way. Better to state it under the table in text form with a specific note (to attract the reader)

R14: We have revised the manuscript to ensure that all graphs meet the minimum standards, including legends and captions. Additionally, we have rewritten the footnote for Table 5 in a clear and structured text format to enhance readability and clarity.

**Discussion**

Q15: In some points you tried to discuss other’s study rather than yours which is not a recommended scientific way of writing.

R15: Thank you for your feedback. We have revised the discussion to ensure that our focus remains on interpreting our own findings while using other studies primarily for contextual support and comparison.

Q16: In some points you said various studies/many studies/different studies but you cited only a single reference.

R16: Thank you for your observation. We have revised the manuscript to ensure consistency between the statements and the number of references cited.

Q17	In some points you said other’s finding low coverage and or quality.

R17: Thank you for your feedback. We have revised the manuscript to clarify that we were comparing effective coverage with crude coverage, rather than simply referring to low coverage in other findings.

Q18: Why preferred to report or compare and contrast in this way?

R18: Thank you for your question. We chose this approach to highlight the difference in the coverage. However, we have revised the manuscript to improve the way we report and compare findings, ensuring that the contrasts and similarities are quantitatively measured and clearly presented for better reader interpretation.

Q19: Why didn’t you compare and contrast in a quantitative way (or why didn’t you show their difference and or similarity rather than concluding others?) which is measurable to judge for the readers. It even difficult to conclude other’s finding

R19: Thank you for your valuable feedback. We have revised the manuscript to include a quantitative comparison where applicable, showing measurable differences and similarities rather than making generalized conclusions. This revision ensures a more objective and data-driven comparison, making it easier for readers to assess the findings in relation to other studies.

Q20: You discussed with a study done on counseling quality which is not similar with yours.

R20: Thank you for your observation. We have revised the discussion to ensure that comparisons are made with studies that are more directly related to our research focus.

Q21: There is an idea which says “Moreover, Facilities in urban areas had higher quality (68.54%). ” Is it your finding or others’? Whether it is your or others’, it is not a recommended scientific ways of writing.

R21: Yes, this is our finding, and we have revised it as follows:

*“The quality of modern contraception services varied across regions, ranging from 73.69% in Addis Ababa to 58.81% in Gambela, indicating regional disparities. This finding is consistent with previous study that reported regional differences in service quality, with scores ranging from 28% in Amhara to 48.7% in Addis Ababa (44). Comparatively, a study conducted in Dire Dawa reported that only 36.6% of women received good-quality family planning services, highlighting a significant gap in service provision (43). Our study further revealed that urban health facilities had a higher quality score (68.54%). This finding aligns with previous research conducted in Ethiopia, which reported that facilities in urban areas (38.0%) were more likely to provide better-quality family planning services than those in rural settings (33.5%) (24). These disparities may be due to the higher employment of experienced personnel in urban areas, better infrastructure, and easier access to health supplies (45). Additionally, higher literacy levels enhance client-provider interactions, while greater economic stability, education, and healthcare access further improve service quality and utilization in urban settings (46).” (Page 28 line 10-22)*

**Conclusion**

Q22: “The study found that, Ethiopia had a poor effective coverage of modern contraceptives services”. This idea is a copy paste of the first sentence.

R22: Thank you for your valuable feedback. We have revised the conclusion statement.

Q23: “Somali and Afar regions had less effective coverage of modern contraceptive service”. This is also repeated

R23: We appreciate your insight and have now removed the repeated sentence.

Q24: “Policymakers and responsible bodies should focus on improving the quality of the facilities in rural areas and the facilities in Gambela, Somali and Afar” This is also repeated

R24: We appreciate your insight and have now removed the repeated sentence.

**Reviewer 2**

Q1: Is it Rhythm?

R1: Thank you for your comment. The correct spelling is 'rhythm,' which refers to the family planning method based on tracking the menstrual cycle. We have corrected it.

Q2: Implant, diaphragm and some other contraceptives categorized as modern methods too.

R2: Thank you for your valuable feedback. We agree with you that methods such as implants, diaphragms, and others are also categorised as modern contraceptive methods. We appreciate your insight and have now incorporated them into the background. We have revised the statement as follows:

“*Family planning methods fall into two categories: traditional methods, such as the rhythm method and withdrawal, and modern methods, which include hormonal methods (such as implants, oral contraceptive pills, and injectables), barrier methods (such as condoms and diaphragms), long-acting reversible contraceptives (such as intrauterine devices), permanent methods (such as sterilization), and emergency contraception.” (Page 4 line 9-12)*

Q3: Based on International agreements like ICPD PoA, family planning main goal is maternal and child health. It is not for population control.

R3: Thank you for your insightful comment. We acknowledge that, based on international agreements like the ICPD PoA, family planning primarily aims to improve maternal and child health rather than control population growth. we have revised the statement to reflect this perspective more accurately.

*“The International Conference on Population and Development (ICPD) Programme of Action (PoA)(1994) emphasizes that family planning is not just a demographic tool but a fundamental right that enhances maternal and child health. It helps reduce maternal mortality by preventing unintended pregnancies, improves child health through optimal birth spacing, empowers women with reproductive choices, and promotes gender equality by supporting education and economic opportunities.” (Page 4 line 4-9)*

Q4: I think traditional methods category needs to be separated from no use.

R4: Thank you for your comment. In our analysis, we use current contraceptive use (only modern contraceptive), we followed the EDHS classification, where only modern contraceptive users (V313 = 3) are included in the numerator. As per the DHS guide, women using traditional methods are included in the denominator. This ensures consistency with DHS methodology and allows for comparability across studies. However, we acknowledge the distinction between traditional method users and those not using any method and will clarify this in the manuscript if needed.

Q5: What is the definition of low in this study?

R5: In this study, 'low' is used in a relative sense, indicating that the effective coverage of family planning services is significantly lower when compared to its crude coverage. We highlight that the effective coverage of modern contraceptive services is significantly lower than the crude coverage at the national level, with crude coverage at 30.27% (95% CI: 28.93–31.62) and effective coverage at 20.41% (95% CI: 19.54–21.28).

Q6: Also, quality of service is related to the client and service provider interaction. In urban area, with usual higher literacy among community, the interaction could be better. Also, the SDH need to be considered.

R6: Thank you for your insightful comment. We agree that the quality of service is influenced by client-provider interactions, which may be enhanced in urban areas due to higher literacy levels. Additionally, we acknowledge the importance of considering social determinants of health (SDH) in understanding these disparities. We have revised the text accordingly to incorporate these aspects.

“*These disparities may be due to the higher employment of experienced personnel in urban areas, better infrastructure, and easier access to health supplies (44). Additionally, higher literacy levels enhance client-provider interactions, while greater economic stability, education, and healthcare access further improve service quality and utilization in urban settings (45).” (Page 28 line 18-22)*

Q7: Is it possible to skip “with higher”?

R7: Sorry for the confusion. It was a mistake, and we have revised it accordingly. Thank you for your feedback.

Q8: This is not a good comparison. NGO and Public sector routinely has significant differences.

R8: We appreciate your valuable input. We have revised the statement by comparing government and private facilities as follows:

“*Our study found that government facilities provided higher-quality modern contraceptive services (68.3%) compared to private facilities (58.11%). This finding is consistent with previous research conducted in Ethiopia, which reported that public facilities offered slightly better service quality than private facilities, with a quality score of 34.5% in public facilities compared to 32.9% in private ones (24). One possible explanation for the higher quality of services in government facilities is that they are often better integrated into national healthcare programs, receiving continuous training, resources, and monitoring to ensure adherence to service quality standards (46, 47).”* *(Page 28 line 22-30)*

Q9: Is it decrease?

R9: We appreciate your attention to detail. The word "decrees" has been corrected to "decrease" to ensure accuracy and clarity in the manuscript. Thank you for your valuable feedback.

Q10: What was definition of low in that study?

R10: Thank you for your valuable feedback. We have revised the manuscript to explicitly clarify that our comparison focuses on effective coverage relative to crude coverage. When we describe coverage as "low," we are referring to instances where the effective coverage is significantly lower than the crude coverage, highlighting gaps in service quality and utilization.

Q11: Actually, I think even in the case of receiving information on the provision of contraceptives through pharmacies, you need a different checklist for the infrastructure and equipment. So, the process needs to be somehow a sophisticated one.

R11: Thank you for your insightful comment. We agree that assessing contraceptive provision through pharmacies would require a different checklist for infrastructure and equipment. Given the limitations of the EDHS 2019 dataset, we were unable to include such details in our analysis. However, we acknowledge that a more sophisticated approach would be necessary for a comprehensive evaluation, and we have noted this as a limitation in our study.

Q12: Please provide a definition for poor.

R12: Thank you for your feedback. We have revised the manuscript to clarify that we were comparing effective coverage with crude coverage.

*“The effective coverage of modern contraceptive services was significantly lower than the crude coverage in Ethiopia. Addis Ababa, western Amhara, northeast Benishangul-Gumuz, northern SNNP, eastern SNNP and northern and central Oromia had highest effective coverage of modern contraceptive. On the other hand, Afar, western Gambela, western Oromia, western Benishangul-Gumuz, Dire Dawa, Harari and Somali had the lowest effective coverage. To enhance equitable access, policymakers should prioritize interventions such as improving healthcare infrastructure and strengthening service delivery to improve modern contraceptive coverage and quality in underserved areas.” (Page 29 )*

Q13: This sentence repeated. Please remove.

R13: Thank you for your valuable feedback. We appreciate your insight and have now removed the repeated sentence.

**Journal Requirements:**

When submitting your revision, we need you to address these additional requirements.

1. Please note that your Data Availability Statement is currently missing [the repository name and/or the DOI/accession number of each dataset OR a direct link to access each database]. If your manuscript is accepted for publication, you will be asked to provide these details on a very short timeline. We therefore suggest that you provide this information now, though we will not hold up the peer review process if you are unable.
   Response: Thank you for your request. We have now revised the data availability statement to reflect that the data can be requested from a third party (demographic and health survey program (<https://dhsprogram.com/>).

   2. Your ethics statement should only appear in the Methods section of your manuscript. If your ethics statement is written in any section besides the Methods, please delete it from any other section.
   Response: Thank you for your guidance. We have removed the ethics statement from all sections except the Methods, as requested.

3. We note your response regarding your map images:

Please note that this does not comply with PLOS's CC-BY 4.0 License, which states that published images may be re-used for any purpose, including commercial use.
As a result, if you would like to proceed with these images, we require specific consent from the copyright holder to publish these images in PLOS ONE, under the CC BY 4.0 license. To seek permission from the copyright owner to publish your map figures under the specific Creative Commons Attribution License (CCAL), CC BY 4.0, please contact them with the following text and PLOS ONE Request for Permission form (<https://journals.plos.org/plosone/s/file?id=c4ff/Content%20Copyright%20Permission%20Form.pdf>):
“I request permission for the open-access journal PLOS ONE to publish XXX under the Creative Commons Attribution License (CCAL) CC BY 4.0 (<http://creativecommons.org/licenses/by/4.0/>). Please be aware that this license allows unrestricted use and distribution, even commercially, by third parties. Please reply and provide explicit written permission to publish XXX under a CC BY license.”

Please upload the granted permission to the manuscript as an other file. In the figure caption of the copyrighted figure, please include the following text: “Republished from [ref] under a CC BY license, with permission from [name of publisher], original copyright [original copyright year].” Please note that RightsLink permission forms often impose use restrictions that are incompatible with our CC BY 4.0 license, and we are therefore unable to accept these permissions. For this reason, we strongly recommend contacting copyright holders with the PLOS ONE Request for Permission form.

Response: Thank you for your feedback. We understand the licensing requirements for publication under the CC BY 4.0 license. To comply with this, we have replaced the original maps with new ones sourced from a freely available and openly licensed source: [Humanitarian Data Exchange (HDX)](https://data.humdata.org/faqs/licenses). The shapefiles used are under a license that permits reuse, including for commercial purposes, which aligns with PLOS ONE’s requirements. We have also updated the figure captions and map sources accordingly. The following link is about the license information for the base shapefile. Licence information: <https://data.humdata.org/faqs/licenses>
