# Supplementary material for: Mapping the effective coverage of modern contraceptive services in Ethiopia
Source: PLoS One. 2025 Jul 7;20(7):e0327581. doi: 10.1371/journal.pone.0327581 (PMC12233249; doi:10.1371/journal.pone.0327581)
Supplement: Attachment — Submitted filename: A_point_by_point_response_auresp_3.docx [file pone.0327581.s009.docx]

**Point by point responses**

We sincerely appreciate the valuable feedback and constructive comments provided by the editor. We have carefully revised the manuscript.

**Editor Comment**

Q1: "Patient and public involvement" is not typically part of the journal's criteria for manuscripts of this type, as the study did not include active participation from patients or the public, despite the obvious relevance of its findings to them.

R1: Thank you for your observation. As the study did not include active participation from patients or the public, we have removed the "Patient and Public Involvement" section from the manuscript to align with the journal’s criteria.
